# Analysis of Program Activities to Develop Forest Therapy Programs for Improving Mental Health: Focusing on Cases in Republic of Korea

**DOI:** 10.3390/healthcare13070760

**Published:** 2025-03-28

**Authors:** Gayeon Kim, Sinae Kang, Kyungsook Paek, Neeeun Lee, Gyeongmin Min, Youngeun Seo, Sooil Park, Seyeon Park, Hyoju Choi, Saeyeon Choi, Pyeongsik Yeon

**Affiliations:** 1 Graduated Department of Forest Therapy, Chungbuk National University, Cheongju 28644, Republic of Korea; rkdus6520@naver.com (G.K.); sinae375@chungbuk.ac.kr (S.K.); ksbaek75@chungbuk.ac.kr (K.P.); share1227@chungbuk.ac.kr (N.L.); akwjs5019@chungbuk.ac.kr (G.M.); youngeun3311@chungbuk.ac.kr (Y.S.); wce6564@chungbuk.ac.kr (S.P.); 0291psy@chungbuk.ac.kr (S.P.); snoopy@chungbuk.ac.kr (S.C.); 2 Department of Forest Sciences, Chungbuk National University, Cheongju 28644, Republic of Korea; hin0358@chungbuk.ac.kr

**Keywords:** forest therapy, depression, anxiety, stress, program activity, mental health, Republic of Korea

## Abstract

This study aims to provide foundational data for developing forest therapy programs to improve mental health conditions by reviewing program development studies, casebooks published by governmental organizations, and existing programs conducted in forest therapy settings and analyzing the status and activities of programs from various disciplines in Korea and abroad. During the collection phase of mental health improvement programs, studies that were not related to depression, anxiety, or stress, or were not relevant to program development, were excluded. Additionally, studies that did not include detailed program plans were omitted from the activity analysis. The literature review focused on academic articles and theses published between 1990 and 2023, identifying 403 relevant studies. Casebooks were collected from three domains, while forest therapy programs were obtained from three forest welfare organizations. These programs were categorized/analyzed based on their general characteristics, structure, and activity content. The findings indicated that research on psychotherapy (forest therapy) programs peaked between 2010 and 2014 (2020 and 2023) in casebooks and field settings. Programs documented in the literature commonly involved 11–20 sessions lasting 60–90 min, whereas casebooks and field programs typically featured fewer than five sessions, each lasting over 180 min. Nineteen purpose-driven and 40 practical activities were identified, providing a theoretical basis for developing forest therapy programs tailored toward alleviating depression, anxiety, and stress. The proposed activities and elements can support the diversification and professionalization of forest therapy programs, fostering more effective and specialized approaches to improve mental health.

## 1. Introduction

The global prevalence and severity of mental health disorders are increasingly recognized, with depression, anxiety, and stress emerging as key indicators of mental health. When left untreated, these conditions can have profound negative consequences [1]. According to the WHO (2023) [2], approximately 5% of adults worldwide experience depression, with over 700,000 people dying by suicide each year. Depression is characterized by persistent feelings of fatigue, loss of pleasure, irritability, or anger, which lead to significant emotional and physical distress. If left untreated, worsening mental health can result in diminished motivation, dependence on substances such as alcohol, or, in extreme cases, an increased risk of suicide [3].

Depression and anxiety are closely interconnected and often co-occur [4]. It is estimated that about 85% of individuals with depression also experience severe anxiety symptoms, which complicates treatment and prolongs recovery [5]. Allen et al. (1995) [6] define anxiety as feelings of fear and unpleasantness, often arising from the anticipation of perceived danger. In severe cases, anxiety can escalate to panic attacks—acute episodes of extreme fear that can mimic life-threatening conditions such as heart attacks or suffocation. Effective strategies to mitigate such episodes are crucial to safeguarding individuals in threatening situations [7].

Stress, which is intricately linked to depression and anxiety, arises from various factors such as work, academic pressure, and interpersonal relationships. Chronic or severe stress can deplete mental and physical resources, leading to “exhaustion” [8]. Stress has been shown to lead to depression [9] and anxiety [10]. These findings highlight the multidisciplinary interest in stress, encompassing clinical psychology, sociology, psychiatry, and public health [11].

Treatment for mental health disorders typically includes both pharmacological and non-pharmacological approaches. While medication remains a mainstay, non-pharmacological interventions have gained traction for populations where medication may not be viable. Various strategies, including mindfulness [12,13], yoga [14,15], exercise [16,17], and breathing techniques [18], have demonstrated efficacy in reducing the symptoms of depression, anxiety, and stress.

Recently, forest therapy has emerged as a promising non-pharmacological approach. It is defined as activities that utilize natural elements such as scents and landscapes to enhance immune function and promote health [19]. In Korea, the Forest Rehabilitation Project carried out between 1973 and 1987 led to an expansion of forested areas. As the scale of forests increased, interest grew in utilizing forest resources to promote public health, leading to the enactment of the FOREST WELFARE PROMOTION ACT in 2015. Following this legislation, the Korea Forest Welfare Institute was established, and research in the field of forest welfare has since diversified [20]. A literature review of forest therapies in Korean adults finds that they can physiologically reduce stress, alleviate depressive symptoms, and improve overall mental health [21]. Forest therapy camps have also been shown to reduce anxiety and depressive symptoms [22], and forest therapy activities have been found to reduce depression and anxiety levels [23], demonstrating the positive effects on facilitating recovery from mental disorders.

As research on forest therapy continues, systematic reviews and meta-analyses have provided further evidence of its effectiveness. Lee et al. (2017) [24] identified forest therapy as an effective intervention for reducing depression in adults, highlighting the need for studies on its long-term effects. Similarly, Yeon et al. (2021) [4] emphasized forest therapy as a preventive and therapeutic non-pharmacological approach for managing depression and anxiety, calling for further exploration of optimal forest characteristics and structured activities. Zhang et al. (2023) [25] found that forest therapy programs significantly alleviated psychological stress, suggesting their potential integration into stress-reduction initiatives.

As the benefits of forest therapy are increasingly recognized, the need for programs tailored to specific populations has also become evident. To address this, Kim (2016) [26] analyzed preferences for forest therapy programs based on stress levels. The study found that individuals with high stress levels preferred cognitively focused programs, such as professional coaching, stress-related lectures, and interpersonal communication workshops. Conversely, those in the low-stress group showed a higher preference for sensory-focused activities, including breathing techniques and exercises; leisurely forest walks (with shoes); listening to flowing water; viewing landscapes; and engaging in forest bathing, air bathing, or sunbathing. These findings underscore the importance of identifying and designing activities that are suited to the needs of each participant. Chae et al. (2020) [27] demonstrate that applying a combination of elements tailored to the characteristics of specific conditions maximizes the effectiveness of forest therapy. Their findings suggest that multi-component programs, such as forest walking, meditation, and horticultural therapy, are particularly suitable for patients. To develop effective forest therapy programs for improving mental health disorders, it is essential to assess participants’ characteristics and needs and provide therapeutic activities that align with them.

Therefore, this study aims to collect programs from various fields designed to address mental health conditions (depression, anxiety, and stress), analyze their general and structural characteristics, and identify and present the activities utilized within these programs. The findings are expected to provide valuable resources for developing forest therapy programs tailored toward improving depression, anxiety, and stress in the future.

## 2. Materials and Methods

### 2.1. Research Design

**Figure 1:**
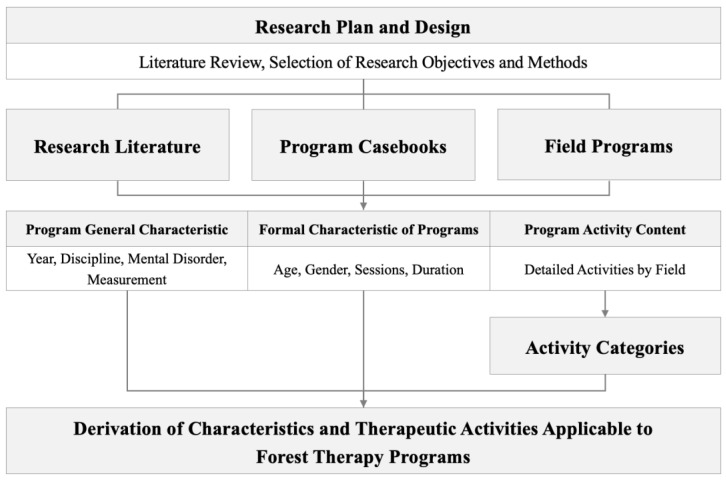
Research design.

In this study, research literature, program casebooks, and field programs were collected to analyze programs developed for improving mental disorders. Subsequently, general and formal characteristics were analyzed, and program activities were derived. The derived program activities were then categorized through discussions among six researchers (Figure 1).

### 2.2. Program Search

This study collected data from the literature, program casebooks, and field programs to investigate interventions aimed at improving mental health conditions, specifically depression, anxiety, and stress.

#### 2.2.1. Research Literature

To explore programs across various fields, academic articles and dissertations related to forestry, psychology, healthcare and nursing, exercise and rehabilitation, social welfare, horticultural therapy, and therapeutic farming were selected. A systematic review of literature databases and keywords for each field was conducted, with four researchers collaborating to finalize the databases and search terms. The search covered studies published from January 1990 to December 2023 in databases such as Scopus, PubMed, MEDLINE, Web of Science, EMBASE, CINAHL (Cumulative Index to Nursing and Allied Health), Research Information Sharing Service (RISS), DBpia, ScienceON, and the National Assembly Library. The search was restricted to studies published in English or Korean. Details of the search terms are provided in Appendix A, Table A1, and Table A2.

#### 2.2.2. Program Casebook

Casebooks from Republic of Korea across fields such as forestry, psychology, healthcare and nursing, exercise and rehabilitation, social welfare, horticultural therapy, and therapeutic farming were reviewed, and their issuing organizations were identified. After reviewing the content, six researchers finalized the selection of casebooks and institutions from three fields: forestry, healthcare, and therapeutic farming. The selected casebooks are listed in Table 1.

#### 2.2.3. Forest Field Program

A field case study was conducted to collect information on mental health improvement programs being implemented in Republic of Korea’s forest environments. The study selected seven national forest welfare institutions under the Korea Forest Welfare Institute, which were operating the 2024 “Mental Health (Stress) Recovery Support Program”. Additionally, 28 forest welfare companies that had contracts with the Korea Forest Welfare Institute for forest therapy programs as of 2023 were included. Each institution and company was contacted to confirm whether they were conducting programs and whether detailed program plans could be provided. Institutions and companies that were not running programs at the time of data collection or could not provide detailed program plans were excluded from the study. Ultimately, programs from three forest welfare institutions were included in the analysis.

### 2.3. Exclusion Criteria

Seven exclusion criteria were used to select data for mental illness recovery programs, and papers that did not include detailed plans to identify activities were excluded because detailed activities of the program had to be extracted. In this study, the data were extracted by checking the full text of the data using the following criteria: (1) Full-text files were unavailable; (2) the program was not related to depression, anxiety, or stress; (3) the program was unrelated to program development; (4) the program analyzed the effectiveness of an already developed program; (5) the program was composed solely of educational activities, not therapeutic activities; (6) detailed program plans were unavailable; (7) the program required specialized qualifications for implementation.

### 2.4. Data Extraction

Six researchers (Gayeon Kim, Sinae Kang, Kyungsook Baek, Youngeun Seo, Hyojoo Choi, and Seyeon Park) independently extracted data from the literature, program casebooks, and field programs. Data that met the selection criteria were chosen after a thorough review of the full text. The extracted information included the general characteristics of the programs (year, academic field, type of mental health condition, and measurement tools), formal program characteristics (participant age, participant gender, number of sessions, and duration), and program activity details. As for the contents of the program activities, activities were extracted by checking the program’s detailed plan, and activities requiring special qualifications were excluded in the program’s progress. After extracting and writing the detailed activities presented in the paper, the researchers reviewed the intersection activities and used methods such as categorizing and citing the criteria of each academic field to exclude subjective opinions of the researchers. Discrepancies during data extraction and analysis were resolved through discussion among the researchers.

## 3. Results

### 3.1. Search Results

Programs aimed at mental health recovery were collected from research literature, program casebooks, and forest field programs. In the thesis extraction process, a large number of studies that did not include detailed plans for program activity analysis were eliminated. A total of 403 studies were identified, including 20 international and 383 Korean publications. Fifty-five programs were extracted from the program casebook, and nine programs were extracted from the forest field programs.

#### Literature Search

**Figure 2:**
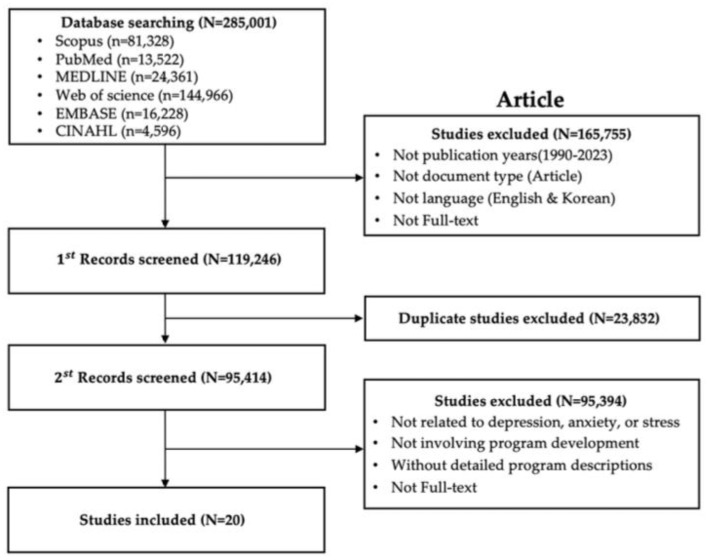
Flowchart of study selection (1).

Figure 2 shows the process of selecting 20 international articles. The primary search yielded 81,328 articles from Scopus, 13,522 from PubMed, 24,361 from MEDLINE, 144,966 from Web of Science, 16,228 from EMBASE, and 4596 from CINAHL. The initial search screened for publication year, language of publication, publication type, and availability of full-text files, resulting in 165,755 exclusions. After excluding 23,832 duplicates, 20 articles were finally selected based on the final selection criteria.

**Figure 3:**
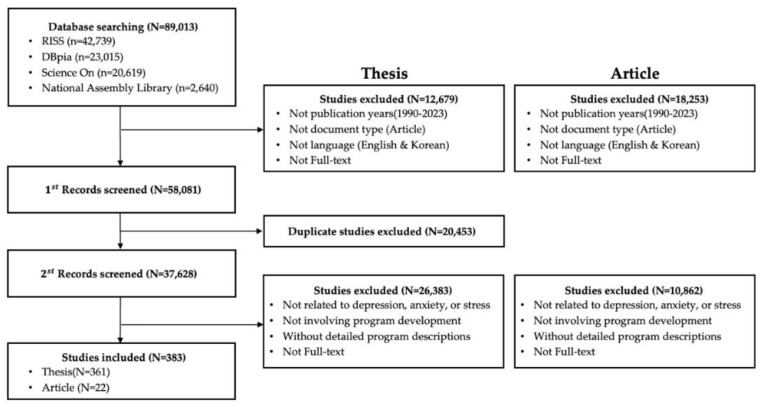
Flowchart of study selection (2).

Figure 3 shows the selection process for 383 Korean articles. The initial search yielded 42,739 articles from RISS, 23,015 from DBpia, 20,619 from Science On, and 2640 from the Library of Congress. The initial search literature was screened for publication year, language of publication, publication type, and availability of full-text files, resulting in 165,755 exclusions. After excluding 20,453 duplicates, 383 articles were finally selected based on the final selection criteria.

### 3.2. Program General Characteristics

The general characteristics of the programs were analyzed by year, discipline, mental disorder, instrument for research literature, program casebooks, and field programs.

Looking at the year of the program, for Korean research literature, 121 (31.6%) of the 383 articles were from 2010–2014, while for international articles, 10 (50.0%) of the 20 articles were from 2020–2023. Among the program casebooks and field programs, 41 (64.1%) of the 64 programs were from 2020–2023 (Table 2).

Looking at the distribution by discipline, in Korean research literature, 252 (65.8%) of the 383 articles were in the field of psychology, while in international articles, 10 (50.0%) of the 20 articles were in the fields of public health, healthcare, and nursing. Among the program casebooks and field programs, 34 (53.1%) of the 64 programs were in the field of forestry (Table 2).

Looking at the type of mental illness targeted by the programs, in Korean research literature, 152 (39.7%) of the 383 articles focused on improving depression, while in international articles, 7 (35.0%) of the 20 articles aimed to reduce stress levels. Among the program casebooks and field programs, 37 (57.8%) of the 64 programs aimed to improve stress (Table 2).

A breakdown of the instruments used to assess program effectiveness in the research literature is presented as follows. The most commonly used tool was the Beck Depression Inventory (BDI), used in 75 studies, followed by the Geriatric Depression Scale (GDS) in 69 studies, the Children’s Depression Inventory (CDI) in 38 studies, the Center for Epidemiological Studies-Depression Scale (CES-D) in 27 studies, and the Self-Rating Depression Scale (SDS) in seven studies.

The State-Trait Anxiety Inventory (STAI) was the most frequently used tool, appearing in 36 studies. Other tools included the Revised Children’s Manifest Anxiety Scale (RCMAS) in 13 studies, the Beck Anxiety Inventory (BAI) in seven studies, the Social Avoidance and Distress Scale (SADS) in four studies, and the Social Anxiety Scale for Adolescents (SAS-A) in four studies.

The Stress Response Inventory (SRI) was used in 23 studies, followed by the Perceived Stress Scale (PSS) in 22 studies, the Korean Preschool Daily Stress Scale (KPDSS) in 19 studies, the Stress Experience Test in 13 studies, and the Way of Coping Checklist (WCC) in 13 studies.

Tools evaluating multiple factors such as depression, anxiety, and stress included the Symptom Checklist-90-Revision (SCL-90-R) in 12 studies, the Profile of Mood States (POMS) in two studies, and the Multiple Affect Adjective Check List (MAACL) in one study. The Korean version of the Depression Anxiety Stress Scale (K-DASS-21) was used in one study.

The following instruments were used to measure effectiveness in casebooks and field programs. Depression was assessed using the Patient Health Questionnaire (PHQ-9) in two programs and the CES-D in one program.

Anxiety was measured using the Generalized Anxiety Disorder-7 (GAD-7) in two programs. For stress, the Worker’s Stress Response Inventory (WSRI) was used in four programs, and a modified version of the SRI was used in one program.

### 3.3. Formal Characteristics of Programs

The formal characteristics of the programs were analyzed in terms of participant age, participant gender, number of sessions, and session duration for research literature, program casebooks, and field programs.

Participant age was classified based on life stages. Among Korean research literature, 164 (42.8%) of the 383 articles targeted children and adolescents, making them the most frequently studied group. In contrast, among international articles, 6 (30.0%) of the 20 articles focused on middle-aged adults. Among the program casebooks and field programs, 21 (32.8%) of the 64 programs did not specify participant age, while 20 (31.3%) targeted young to older adults (Table 3).

Looking at participant gender, among Korean research literature, 236 (61.6%) of the 383 articles targeted mixed-gender groups, while among international articles, 14 (70.0%) of the 20 articles also targeted mixed-gender groups. Among the program casebooks and field programs, 53 (82.8%) of the 64 programs did not specify participant gender, while 11 (17.2%) were designed for mixed-gender groups (Table 3).

The program’s progress session was for the research studies; 186 studies (48.7%) were conducted between 11 and 20 sessions. For international articles, 11 studies (55.0%) were conducted between 5 and 10 sessions. For casebooks and field programs, however, 42 out of 64 programs (65.6%) were conducted in fewer than five sessions (Table 3).

The duration of the program was expressed in minutes. Among Korean research literature, 127 (33.2%) of the 383 articles had sessions lasting between 60 and 90 min. Among international articles, 5 (25.0%) of the 20 articles did not specify session duration, while 4 articles (20.0%) each had sessions lasting between 30 and 60 min and between 60 and 90 min. Among the program casebooks and field programs, 25 (39.1%) of the 64 programs had sessions lasting 180 min or longer (Table 3).

### 3.4. Program Activity Content

The activities extracted from programs across various fields (forestry, psychology, healthcare and nursing, exercise and rehabilitation, social welfare, and horticultural therapy and therapeutic farming) were categorized based on the following two criteria: (1) activities that have a purpose and are developed around that purpose, and (2) activities that are not purpose-driven and can be used in a variety of ways. After categorizing the activities by purpose, we extracted the detailed activities for each area, as described below.

#### 3.4.1. Purpose-Driven Activities

A total of 19 purpose-driven activities were identified, encompassing 1025 sub-activities. The most frequently observed sub-activity was “Implementing and Evaluating Solutions”, with 151 instances. This was followed by “Recognizing Myself” with 147 sub-activities, “Enhancing Social Skills” with 130 sub-activities, and “Recognizing Emotions” with 94 sub-activities (Table 4).

Table 5 presents the top five purpose-driven activities by frequency. In psychology, “Implementing and Evaluating Solutions” ranked first. In the healthcare and nursing field (research literature), as well as exercise and rehabilitation and therapeutic farming (casebooks and field programs), “Enhancing Social Skills” was the most frequent activity. In social welfare, “Expressing Emotions” ranked first, while in horticulture and therapeutic farming, “Recalling the Past” was the most frequent. For forestry (research literature), “Recognizing Senses” ranked first. In healthcare (casebooks and field programs), “Implementing and Evaluating Solutions” and “Identifying Ways to Cope with and Manage Stress” were tied for first place. In forestry (casebooks and field programs), “Recalling the Past”, “Recognizing Oneself”, and “Expressing Stress” were tied for first place.

#### 3.4.2. Practical Activities

A total of 40 practical activities were identified, with a total of 1468 sub-activities. Among these, “Meditation” had the highest number of sub-activities (138), followed by “Art Activities” with 132 sub-activities and “Play” with 122 sub-activities (Table 6).

The top five practical activities identified in programs across various fields are summarized in Table 7. In psychology, art activities ranked first in the research literature. In healthcare and nursing, meditation and laughter activities ranked first. In exercise and rehabilitation, dance activities ranked first. In social welfare, playing ranked first. In horticulture and therapeutic farming, indoor gardening ranked first. In forestry, forest experiences ranked first. For casebooks and field programs, the first-ranked activities were as follows: Crafts and physical relaxation were tied for first in healthcare. In therapeutic farming, cooking activities and indoor gardening were tied for first. In forestry, meditation activities ranked first.

### 3.5. Forest Environment Characteristics

The characteristics of forest environments were analyzed based on programs conducted in forest settings for mental health improvement. A total of 16 programs from research literature and 34 programs from program casebooks and field programs were reviewed. Among these, environmental characteristics were identified in 4 research literature programs and 18 casebook programs. The key forest environment attributes presented in the programs included dominant tree species, slope gradient, elevation, and canopy cover.

The dominant tree species included *Pinus densiflora* (Japanese red pine), *Pinus koraiensis* (Korean pine), *Quercus mongolica* (Mongolian oak), *Quercus variabilis* (Oriental cork oak), *Phyllostachys* spp. (bamboo), *Ginkgo biloba* (ginkgo), *Larix kaempferi* (Japanese larch), *Betula platyphylla* (Asian white birch), *Metasequoia glyptostroboides* (dawn redwood), and *Chamaecyparis pisifera* (sawara cypress), with *Pinus densiflora* being the most frequently mentioned.

The slope gradient varied across programs, with reported values of 8%, 6–11%, 0–15%, and 20%, with 8% being the most common. Elevation ranged from 100–150 m, 100–400 m, 450–630 m, 850 m, and 400–992 m. Canopy cover values were reported as approximately 60%, 65–80%, and 80.1–86.3%.

## 4. Discussion

This study analyzed the characteristics and activities of programs developed to improve depression, anxiety, and stress to inform the development of forest therapy programs for mental health recovery. The key findings are discussed below.

First, regarding the general characteristics of the programs, an analysis of publication years and academic fields revealed that the number of research studies steadily increased from the 1990s, peaking between 2010 and 2014, followed by a decline. For casebooks and field programs, the earliest programs appeared in 2010, with the highest number of programs identified between 2020 and 2023. In terms of academic disciplines, most studies focused on psychology, followed by healthcare and nursing, whereas casebooks and field programs were predominantly forestry-based. This indicates that non-pharmacological approaches have long been explored to improve mental health in the field of psychology, while in forestry, diverse programs for improving mental health began to be developed and implemented after 2010. The rise in group therapy programs targeting depression after 2010 [28] and psychological interventions for anxiety [29] reflect growing research and program development in mental health recovery.

Second, regarding the types of mental health conditions addressed, research studies primarily targeted depression, followed by stress, whereas casebooks and field programs focused mainly on stress. This may reflect the practical challenges of accessing participants with high levels of depression and anxiety in field settings, leading to the development of programs targeting stress reduction. An analysis of the collected research literature revealed that various therapeutic approaches have been developed to reduce depression levels, demonstrating positive effects on participants. These include positive psychology interventions [30,31], art therapy [32,33], cognitive-behavioral therapy (CBT) [34,35], acceptance and commitment therapy (ACT) [36,37], and others. Additionally, to reduce stress levels, programs utilizing positive psychology interventions [38,39], bibliotherapy [40,41], mindfulness meditation [42,43], yoga [44,45], and other approaches have been developed, showing positive effects on stress reduction. Finally, for alleviating anxiety, programs incorporating art therapy [46,47], meditation [48,49], literature therapy [40,50], and other methods have been designed and found to be effective in reducing anxiety levels.

Third, regarding the formative characteristics of the programs, research studies predominantly targeted children and adolescents, while casebooks and field programs focused on young to older adults. In research, studies often developed programs tailored to specific populations, whereas field programs tended to offer more generalized programs for young and older adults. Differences were also observed in the number and duration of sessions across research, casebooks, and field programs. Research studies typically featured 11–20 sessions lasting 60–90 min, while field programs commonly involved fewer than five sessions lasting over 180 min. These differences likely stem from the contrasting characteristics of psychological programs, which were common in research studies, and forestry programs, which were prevalent in casebooks and field programs.

Fourth, program activities were categorized into purpose-driven (19) and practical (40), making for a total of 59 activities. Among these, five activities—recognizing senses; recognizing emotions; recognizing oneself; enhancing social skills; and art activities—were common across all fields; indicating that certain activities are universally applicable despite differences in focus areas. Among purpose-driven activities, “Implementing and Evaluating Solutions” was the most frequently identified. This highlights the importance of teaching participants actionable skills and techniques for mental health recovery, such that participants can apply and refine these methods during the program and in their daily lives. For practical activities, meditation was the most frequently identified. The effectiveness of meditation in reducing depression, anxiety, and stress has been consistently demonstrated [51,52], which explains its widespread inclusion in program designs.

Fifth, given the importance of forest environmental characteristics in developing forest therapy programs for mental health improvement, this study analyzed the environmental attributes of programs conducted in forest settings. However, among 50 forest-related programs, only 22 provided information on forest environmental characteristics, with dominant tree species, slope gradient, elevation, and canopy cover being the most commonly reported attributes.

A review of previous studies revealed that forest walking programs effectively reduced depressive symptoms in adult women [53], while forest therapy programs alleviated stress [54], fatigue, and depression [55] in middle-aged women. Additionally, nature-based stress management programs for university students were found to decrease stress levels [56], and self-directed forest therapy programs effectively reduced stress and depression in university students [57]. Furthermore, forest activities for infants and young children positively impacted stress reduction [58,59,60,61,62], and school forest experience programs were found to lower depression levels in elementary school students [63]. These findings align with the Psycho-Evolutionary Theory (PET), which argues that natural environments have psychologically and physiologically positive effects on humans and help reduce stress [64,65].

Although the effectiveness of forest programs in improving mental health has been well documented, there remains a lack of detailed descriptions of forest environmental characteristics. Consequently, it is difficult to determine whether specific environmental attributes directly contributed to mental health improvements. Moreover, while program casebooks frequently included forest environment descriptions, they lacked efficacy assessments. Therefore, this study cannot conclude that specific forest environmental characteristics directly influenced mental health improvements.

This study collected and analyzed programs developed for mental health recovery to inform the development of forest therapy programs. However, several limitations should be noted.

First, the analysis focused on research studies published between 1990 and 2023 and casebooks and field programs from 2010 to 2024. The results of all 403 extracted papers cannot be presented in a table. Consequently, caution is needed when generalizing the findings.

Second, for program activity analysis, programs without detailed plans were excluded from the analysis when collecting programs. There is a limit to generalization because a large number of academic papers are not presented with a detailed plan; therefore, degree papers published in Korea occupy a large proportion.

Third, six researchers were unable to exclude the subjective opinions of researchers by categorizing the program’s activities, so more objective criteria will be needed in future studies. In addition, because the scope of classification activities is comprehensive, specific activities should be presented in subsequent studies.

Fourth, program activities in various fields were analyzed to provide basic data for developing forest healing programs; however, the analysis of the forest environment was insufficient. Forest-related papers were often excluded because they did not include detailed plans, and most of the papers adopted did not mention the forest environment. When developing forest-based intervention programs, it is crucial to document forest environmental characteristics. Furthermore, future research should conduct impact analyses to determine the extent to which specific environmental attributes contribute to mental health benefits.

## 5. Conclusions

This study collected programs for mental illness recovery in various fields and analyzed program characteristics and activity contents. The conclusions drawn from the findings are presented as follows:

First, the analysis included program elements such as publication year, academic field, mental health condition, measurement tools, participant age, gender, number of sessions, and session duration. By identifying the current state and characteristics of these elements, the findings can guide the development of forest therapy programs tailored to the needs and circumstances of participants.

Second, while some similarities were observed between research studies and casebooks or field programs, the differences were more pronounced. These differences likely reflect the dominant focus of each domain—psychology in the research literature and forestry in casebooks and field programs. This highlights the importance of recognizing the unique characteristics of each field and developing programs that align with their specific objectives when designing forest therapy interventions.

Third, this study aimed to identify a diverse range of activities from programs targeting reductions in depression, anxiety, and stress. More effective programs could be created and provided by selecting activities suited to the specific mental health conditions and symptoms of participants and incorporating activities that address their individual needs.

Fourth, this study is significant in that it identified the characteristics and activities of programs that can aid in the recovery of mental disorders, thereby presenting various therapeutic activities across different fields. However, when developing forest therapy programs, it is essential to integrate forest environmental characteristics into program activities. By analyzing the environmental attributes of the forests where programs are conducted and incorporating therapeutic resources into the activities presented in this study, more effective forest therapy programs can be designed and implemented.

The program characteristics and activities presented in this study are expected to serve as a foundation for developing and providing forest therapy programs tailored to the conditions and symptoms of participants.

## Figures and Tables

**Table 1:** Program casebook list.

Field	Issuing Institution	Casebooks Title
Forestry	Korea Forest Welfare Institute	Forest Welfare Program Casebook Major Programs of Forest Welfare Service in 2018
Healthcare	National Center for Mental Health	Casebook of excellent mental health services in the community Precious time for you and me
Therapeutic farming	National Institute of Agricultural Sciences	Insects, Ecology, and Healing Programs: Let’s Fly with the Tiger Swallowtail Harmony and Rest: Rural Healing Mental Health in Rural Communities for Young Adults
	National Institute of Horticultural and Herbal Science	Psychological horticultural therapy program for people returning to society It’s All Okay”: Horticultural Therapy for Cancer Patient Recovery Management Horticultural Program for Emotional Development of Middle School Students Who Perpetrated School Violence Parent’s Garden for a Happy Child: Plant Growing for Enhancing Parenting Roles Manual for Improving Lifestyle-Related Diseases Gardening Stories for Enjoying Daily Living

**Table 2:** Program general characteristics (year, discipline, mental disorder).

Characteristics	Category	Criteria	Frequency (%)	
			Korean	International
Year	Research literature	1990~1999	4 (1.0)	1 (5.0)
		2000~2004	21 (5.5)	-
		2005~2009	64 (16.7)	-
		2010~2014	121 (31.6)	1 (5.0)
		2015~2019	108 (28.2)	8 (40.0)
		2020~2023	65 (17.0)	10 (50.0)
	Program casebook and field programs	2010~2014	1 (1.6)	-
		2015~2019	17 (26.6)	
		2020~2023	41 (64.1)	
		2024	5 (7.8)	
Discipline	Research literature	Psychology	252 (65.8)	6 (30.0)
		Public health, healthcare, and nursing	31 (8.1)	10 (50.0)
		Exercise and rehabilitation	30 (7.8)	3 (15.0)
		Social welfare	30 (7.8)	-
		Horticulture and Therapeutic Farming	24 (6.3)	1 (5.0)
		Forestry	16 (4.2)	-
	Program casebook and field programs	Healthcare	15 (23.4)	-
		Therapeutic farming	15 (23.4)	
		Forestry	34 (53.1)	
Mental disorder	Research literature	Depression	152 (39.7)	3 (15.0)
		Anxiety	34 (8.9)	2 (10.0)
		Stress	119 (31.1)	7 (35.0)
		Depression, anxiety	37 (9.7)	2 (10.0)
		Depression, stress	31 (8.1)	3 (15.0)
		Anxiety, stress	5 (1.3)	-
		Depression, anxiety, and stress	5 (1.3)	3 (15.0)
	Program casebook and field programs	Depression	2 (3.1)	-
		Anxiety	-	
		Stress	37 (57.8)	
		Depression, anxiety	4 (6.3)	
		Depression, stress	10 (15.6)	
		Anxiety, stress	1 (1.6)	
		Depression, anxiety, and stress	10 (15.6)	

**Table 3:** Formal characteristics of programs (participant age, gender, sessions, duration).

Characteristics	Category	Criteria	Frequency (%)	
			Korean	International
Age	Research literature	Infants and toddlers	11 (2.9)	-
		Children and adolescents	164 (42.8)	3 (15.0)
		Young adults	34 (8.9)	5 (25.0)
		Middle-aged adults	49 (12.8)	6 (30.0)
		Older adults	66 (17.2)	1 (5.0)
		Middle-aged to older adults	19 (5)	1 (5.0)
		Young adults to middle-aged adults	23 (6)	1 (5.0)
		Young adults to older adults	7 (1.8)	2 (10.0)
		Not presented	10 (2.6)	1 (5.0)
	Program casebook and field programs	Infants and toddlers	-	-
		Children and adolescents	9 (14.1)	
		Young adults	2 (3.1)	
		Middle-aged adults	2 (3.1)	
		Older adults	5 (7.8)	
		Infants and toddlers to children and adolescents	1 (1.6)	
		Young adults to older adults	20 (31.3)	
		Middle-aged to older adults	1 (1.6)	
		All ages	3 (4.70)	
		Not presented	21 (32.8)	
Gender	Research literature	Mixed	236 (61.6)	14 (70.0)
		Male	15 (3.9)	-
		Female	103 (26.9)	4 (20.0)
		Not presented	29 (7.6)	2 (10)
	Program casebook and field programs	Mixed	11 (17.2)	-
		Male	-	
		Female	-	
		Not presented	53 (82.8)	
Sessions	Research literature	<5 sessions	12 (3.1)	1 (5.0)
		5–10 sessions	164 (42.8)	11 (55.0)
		11–20 sessions	186 (48.7)	1 (5.0)
		21–30 sessions	9 (2.3)	3 (15.0)
		≥31 sessions	5 (1.3)	3 (15.0)
		Not presented	7 (1.8)	1 (5.0)
	Program casebook and field programs	<5 sessions	42 (65.6)	-
		5–10 sessions	16 (25.0)	
		11–20 sessions	4 (6.3)	
		21–30 sessions	-	
		≥31 sessions	-	
		Not presented	2 (3.1)	
Duration (minutes)	Research literature	<30	5 (1.3)	2 (10.0)
		30–59	107 (27.9)	4 (20.0)
		60–89	127 (33.2)	4 (20.0)
		90–119	96 (25.1)	1 (5.0)
		120–179	28 (7.3)	3 (15.0)
		≥180	6 (1.6)	1 (5.0)
		Not presented	14 (3.6)	5 (25.0)
	Program casebook and field programs	<30	1 (1.6)	-
		30–59	5 (7.8)	
		60–89	3 (4.7)	
		90–119	3 (4.7)	
		120–179	17 (26.6)	
		≥180	25 (39.1)	
		Not presented	10 (15.6)	

**Table 4:** Purpose-driven activities.

No.		Program Areas	Research Literature					Casebooks and Field Programs				Total
	Name of Activity		Psychology	Healthcare and Nursing	Exercise and Rehabilitation	Social Welfare	Horticulture Therapeutic Farming	Forestry	Healthcare and Nursing	Therapeutic Farming	Forestry	
1	Recognize value		13	-	-	-	-	-	-	-	-	13
2	Recognizing senses		11	1	8	6	1	21	4	2	4	58
3	Recognizing emotions		66	4	6	8	2	3	3	1	1	94
4	Expressing emotions		16	2	2	24	3	4	1	-	3	55
5	Recalling the past		29	8	1	15	6	-	3	-	5	67
6	Finding your dream		34	6	-	11	4	1	2	1	3	61
7	Recognizing myself		97	5	8	20	2	4	4	2	5	147
8	Analyzing the problem		19	5	-	-	-	-	-	-	-	24
9	Identifying and planning solutions		16	2	1	2	-	-	-	-	1	22
10	Implementing and evaluating solutions		110	9	8	9	2	3	8	-	2	151
11	Enhancing social skills		65	10	9	23	1	15	3	3	1	130
12	Recognizing thoughts		13	3	2	1	-	1	1	-	-	21
13	Finding ways to cope and manage stress		5	3	-	2	-	-	8	-	-	18
14	Recognizing stress		12	1	1	3	-	3	-	2	1	23
15	Expressing stress		9	-	6	9	-	7	-	-	5	36
16	Cognitive restructuring and awareness		9	1	-	3	-	-	-	-	-	13
17	Identifying strengths and weaknesses		39	2	3	3	1	1	1	-	2	52
18	Becoming aware of the present		9	1	1	-	-	1	-	-	-	12
19	Finding happiness		17	6	-	4	-	-	1	-	-	28
Total			589	68	56	143	22	64	39	11	33	1025

**Table 5:** Rankings of purpose-driven activities by field.

Rank	Research Literature						Casebooks and Field Programs		
	Psychology	Healthcare and Nursing	Exercise and Rehabilitation	Social Welfare	Horticulture Therapeutic Farming	Forestry	Healthcare and Nursing	Therapeutic Farming	Forestry
1	Implementing and evaluating solutions	Enhancing social skills	Enhancing social skills	Expressing emotion	Recalling the past	Recognizing senses	Implementing and evaluating solutions and finding ways to cope with and manage stress	Enhancing social skills	Recalling the past, recognizing myself, expressing stress
2	Recognizing myself	Implementing and evaluating solutions	Recognizing senses, Recognizing myself, finding your dream	Enhancing social skills	Finding your dream	Enhancing social skills		Recognizing senses, Recognizing myself, recognizing stress	
3	Recognize emotions	Recalling the past		Recognizing myself	Expressing emotions	Expressing stress	Recognizing senses, recognizing myself		
4	Enhancing social skills	Find your dream, Finding happiness		Recalling the past	Recognizing senses, recognizing myself, implementing and evaluating solutions	Expressing emotions, recognizing myself			Recognizing senses
5	Strengths and weaknesses Identifying strengths and weaknesses		Recognizing emotions, expressing stress	Finding your dream			Recognizing emotions, recalling the past, enhancing social skills	Recognizing emotions, finding your dream	Expressing emotions, finding your dream

**Table 6:** Practical activities.

No.		Program Areas	Research Literature						Casebooks and Field Programs			Total
	Name of Activity		Psychology	Healthcare and Nursing	Exercise and Rehabilitation	Social Welfare	Horticulture Therapeutic Farming	Forestry	Healthcare and Nursing	Therapeutic Farming	Forestry	
1	Assigning tasks		9	-	4	-	2	-	-	-	-	15
2	Setting rules		4	1	2	2	1	-	-	-	-	10
3	Playing		52	19	9	40	-	-	2	-	-	122
4	Tea ceremony		15	-	-	8	3	1	1	2	4	34
5	Behavior Expressions		9	-	-	5	-	4	-	-	-	18
6	Massage		2	5	-	1	2	2	1	2	6	21
7	Crafts		14	3	-	14	29	10	7	10	3	90
8	Meditation		53	34	13	6	-	7	1	2	22	138
9	Literary activities		17	-	-	7	-	-	-	2	2	28
10	Art activities		63	21	5	20	3	8	4	4	4	132
11	Creating nicknames		9	1	-	3	2	1	-	1	-	17
12	Stretching		5	1	3	1	-	1	-	2	8	21
13	Body relaxation		7	8	5	2	-	-	7	1	1	31
14	Mind-body workouts		3	6	9	-	-	1	-	-	-	19
15	Ice-breaker		34	3	6	4	-	-	1	-	2	50
16	Role play		4	-	-	1	-	1	-	-	-	6
17	Yoga		10	7	8	3	-	1	-	-	2	31
18	Cooking		7	1	-	2	11	2	3	11	1	38
19	Laughter activities		22	34	-	11	-	-	-	-	2	69
20	Music activities		42	19	7	9	-	-	-	-	1	78
21	Self-introduction		18	5	3	9	1	-	-	-	1	37
22	Playing with nature		2	-	-	-	-	19	-	-	4	25
23	Dancing		9	1	39	5	-	-	-	-	-	54
24	Breathing Techniques		2	2	4	6	-	3	3	-	7	27
25	Wrapping up an activity		24	3	4	6	1	-	1	-	-	39
26	Indoor Gardening		8	5	-	8	30	-	2	11	-	64
27	Outdoor garden		12	-	-	-	-	-	-	10	-	22
28	Brain Stimulation exercise		1	3	9	-	-	-	-	-	-	13
29	Forest Experiences		4	1	-	-	3	35	-	2	11	56
30	Insect-mediated activities		-	-	-	-	-	1	-	6	-	7
31	Animal-mediated activities		-	-	-	-	-	2	-	3	-	5
32	Creative activities		11	1	2	9	-	5	1	-	-	29
33	Hydrotherapy		-	-	-	-	2	2	-	1	5	10
34	Forest Walking		-	-	-	-	1	8	-	2	14	25
35	Forest writing		-	-	-	-	-	3	-	-	8	11
36	Forest Workout		-	-	-	-	-	-	-	-	16	16
37	Storytelling		-	-	-	-	-	6	1	-	2	9
38	Video Media Activity		-	5	-	-	1	-	2	-	1	9
39	Exercise		-	10	24	-	-	1	-	-	4	39
40	Healing Equipment Experience		-	-	-	-	-	-	-	-	3	3
Total			472	199	156	182	92	124	37	72	134	1468

**Table 7:** Rankings of practical activities by field.

Rank	Research Literature						Casebooks and Field Programs		
	Psychology	Healthcare and Nursing	Exercise and Rehabilitation	Social Welfare	Horticulture Therapeutic Farming	Forestry	Healthcare and Nursing	Therapeutic Farming	Forestry
1	Art activities	Meditation, laughter activities	Dancing	Playing	Indoor Gardening	Forest Experiences	Crafts, physical relaxation	Cooking Activities, Indoor Gardens	Meditation
2	Meditation		Exercise	Art activities	Crafts	Playing with nature			Forest Workout
3	Playing	Art activities	Meditation	Crafts	Cooking activities	Crafts	Art activities	Crafts, outdoor gardening	Forest Walking
4	Music activities	Play, music, and activities	Play, mind-body workout, brain-activating exercise	Laughter activities	Tea ceremonies, art activities, and forest experiences	Art activities, forest walks	Cooking Activities, Breathing Techniques		Forest Experiences
5	Icebreaking			Music, Introduce Yourself, Create				Insect-borne activity	Stretching, Forest writing

## Data Availability

The data presented in this study are available on request from the corresponding author. The data are not publicly available due to privacy.

## Appendix A

**Table A1:** Search terms used in each database (1).

Category	Keywords
Intervention	“forest*” OR “forest exposure” OR “forest bathing” OR “forest healing” OR “forest therap*” OR “shinrin-yoku” OR “urban forest*” OR “green space” OR “greenspace” OR “urban green*” OR “green exercise” OR “tree*” OR “garden*” OR “park” OR “parks” OR “landscape*” OR “horticulture*” OR “horticultural*” OR “wood*” OR “natural environment*” OR “green infrastructure*” OR “greenness” OR “nature-based” OR “outdoor*” OR “recreation” OR “forest experience” OR “forest activity” OR “forest adventure” OR “nature” OR “natural activity” OR “forest walking” OR “Hospitals” OR “Centers” OR “Therap*” OR “Treatment” OR “Mentali*” OR “art therap*” OR “experiment” OR “service” OR “hydro therapy” OR “training” OR “Exercise” OR “Recovery” OR “intervention”
Intervention	“program”
Effects	“depress*” OR “depression” OR “depressive disorder” OR “anxiety*” OR “anxiety disorder” OR “Hamilton Rating Scale s for Depression” OR “Hamilton Depression Rating Scale” OR “Beck Depression Inventory” OR “Hospital Anxiety and Depression Scale” OR “Montgomery-Asberg Depression Rating Scale” OR “ZungSelf-Rating Depression Scale” OR “Stress” OR “cortisol” OR “stress response index” OR “mental health”
Effects	“Effect” OR “Development” OR “Impact”

**Table A2:** Search terms used in each database (2).

Category	Keywords
Intervention	“숲” OR “산림” OR “자연” OR “산림치유” OR “도시숲” OR “치유” OR “체험” OR “숲놀이” OR “삼림욕” OR “테라피” OR “활동” OR “경험” OR “요법” OR “산림운동” OR “숲치유” OR “산림교육” OR “경관” OR “산림휴양” OR “센터” OR “치료 “ OR “환자” OR “중재” OR “비약물적중재” OR “원예” OR “치유정원” OR “식물재배” OR “개입” OR “미술치료” OR “상 담” OR “음악치료” OR “이야기치료” OR “처치” OR “행동” OR “집단프로그램” OR “사업” OR “서비스” OR “심리” OR “ 자조모임” OR “지역사회통합돌봄” OR “신체활동” OR “재활운동”
Intervention	“프로그램”
Effects	“우울” OR “불안” OR “스트레스” OR “질환자” OR “정신보건” OR “정신건강”
Effects	“영향” OR “효과” OR “개발”
